# A Dual-Functional Bi_3_TiNbO_9_/Bi_2_MoO_6_ Heterojunction for Simultaneous Environmental Remediation and CO_2_ Photoreduction

**DOI:** 10.3390/nano15241903

**Published:** 2025-12-18

**Authors:** Reshalaiti Hailili, Yiming Gan

**Affiliations:** Beijing Key Laboratory of Heat Transfer and Energy Conversion, Beijing University of Technology, Beijing 100124, China; ganyiming@emails.bjut.edu.cn

**Keywords:** Bi_3_TiNbO_9_/Bi_2_MoO_6_ (BTNO/BMO), heterojunction, layered structure, pollutant treatment, CO_2_ reduction

## Abstract

The development of versatile photocatalysts is crucial for comprehensive solutions to the intertwined challenges of the energy crisis and environmental pollution. This study presents a novel Bi_3_TiNbO_9_/Bi_2_MoO_6_ (BTNO/BMO) heterojunction fabricated via a solvothermal method. Advanced characterization techniques verified the successful synthesis of the as-integrated BTNO/BMO heterostructure. The BTNO/BMO composite exhibited superior performance in multiple applications: efficient degradation of tetracycline reaching 90.2%, removal of gaseous nitric oxide (NO), and photocatalytic reduction of carbon dioxide (CO_2_) to carbon monoxide (CO) with a yield of 51.3 μmol·g^−1^. The constructed Type-II heterojunction demonstrated a remarkable ability to suppress charge recombination, thereby significantly enhancing the photocatalytic activity. This work highlights the dual-functional capability of the BTNO/BMO heterojunction for simultaneous environmental purification and fuel production, providing a promising material platform and a strategic design concept for sustainable technological development.

## 1. Introduction

With the rapid advancement of industrialization and urbanization, the challenges of the energy crisis and environmental pollution have become increasingly severe, driving an urgent need for sustainable technological solutions [1,2]. Tetracycline (TC), a commonly used antibiotic, is widely employed to treat various diseases. However, due to its poor absorption by the human body, most of it is excreted into aquatic environments through feces, urine, and other bodily fluids. Bacteria in water contaminated with tetracycline gradually develop resistance to the drug, disrupting aquatic ecosystems, inducing drug-resistant bacteria, and increasing the risk of human infection [3,4]. In addition, the accumulation of organic pollutants in water bodies and the excessive emission of atmospheric pollutants such as nitrogen oxides (NOx) and carbon dioxide (CO_2_) pose significant threats to ecosystems and human health [5,6,7]. While conventional technologies such as selective catalytic reduction (SCR) for NOx and carbon capture and storage (CCS) for CO_2_ have been widely applied, they often suffer from limitations including high energy consumption, operational complexity, and potential secondary pollution [8,9]. In this context, semiconductor-based photocatalysis has emerged as a promising green technology capable of utilizing solar energy to degrade pollutants and convert CO_2_ into value-added chemicals, enabling a “waste-to-resource” goal to be achieved under mild conditions [10,11].

Many cutting-edge investigations have been devoted to photocatalyst design and efficiency improvements to tackle both the energy crisis and environmental pollution [12,13]. However, the widespread application of photocatalysis is still hindered by the limited performance of single-component catalysts, which often exhibit rapid recombination of photogenerated charge carriers, narrow light absorption ranges, and insufficient redox capability [14]. Among various semiconductor materials, bismuth-based photocatalysts have attracted considerable attention due to their unique layered architecture, tunable electronic bandgaps, and high chemical stability [15,16]. For instance, Bi_3_TiNbO_9_ (BTNO), a representative Aurivillius-type bismuth-based photocatalyst, consists of alternating layers of [Bi_2_O_2_]^2+^ and perovskite-like [BiTiNbO_7_]^2−^ slabs. In such a unique layered structure, intra-layer strong electrostatic interactions generate a spontaneous polarization field that effectively promotes separation of photoinduced electron–hole pairs while suppressing carrier recombination. Its high anisotropy further positions it as an ideal material for designing highly efficient photocatalysts [17,18,19,20]. On the other hand, bismuth molybdate (Bi_2_MoO_6_, BMO), another Aurivillus-phase material, exhibits excellent visible-light absorption and a tendency to form two-dimensional nanosheets, making it suitable for constructing interfacial heterojunctions [21,22,23,24]. Beyond traditional binary heterojunctions, attention has gradually shifted toward ternary and even higher-order heterojunctions combining BMO with other materials. When two or more semiconductor catalysts are combined, Fermi levels with differing potentials migrate toward each other and eventually stabilize at the same position. Simultaneously, photogenerated electrons and holes migrate across the material interface, forming thin layers of positive and negative charges [25,26,27]. Since single-component BTNO or BMO still struggles to fully overcome the inherent limitations of semiconductor photocatalysts such as low solar-light conversion, rapid charge-carrier separation and the ambiguous surface-interface reaction mechanism, constructing heterojunctions, particularly tightly contacted II-type or Z-type structures, has proven to be an effective strategy for enhancing light absorption, promoting charge separation, and maintaining strong redox potentials [28,29,30].

In this study, a novel BTNO/BMO heterojunction was designed and synthesized. By coupling BTNO with BMO, we aim to achieve the synergistic effects of (i) extended light absorption range, (ii) facilitated separation and migration of photogenerated carriers driven by a built-in electric field, and (iii) preservation of strong redox ability for simultaneous oxidation and reduction reactions. The microstructure, optical properties, and band alignment of the BTNO/BMO heterojunction were systematically investigated. The photocatalytic performance was further evaluated across multiple applications, including tetracycline degradation, NO removal, and CO_2_ reduction. Results indicate that the heterojunction exhibits significantly enhanced activity compared to the individual components, which can be attributed to the formation of a Type-II charge transfer pathway that effectively concentrates electrons and holes at respective active sites. This work not only provides a high-performance composite photocatalyst for integrated environmental remediation and energy conversion but also offers deeper insight into the rational design of multifunctional heterojunction systems for sustainable photocatalytic applications.

## 2. Experimental Section

### 2.1. Preparation of Photocatalysts

**Materials.** The employed raw materials e.g., Bi(NO_3_)_3_·5H_2_O and Na_2_MoO_4_·2H_2_O were purchased from Guangfu Institute of Fine Chemical, Ch (Tianjin, China), Nb_2_O_5_ and NaOH were purchased from Shanghai Aladdin Bio-Chem Technology Co., Ltd. (Shanghai, China). The titanium isopropoxide (C_16_H_36_O_4_Ti) and ethylene glycol were purchased from Anhui Senrise Technology Co., Ltd. (Hefei City, Anhui, China). All the reagents were of analytical grade purity and used without further purification.

**Synthesis of Bi_3_TiNbO_9_:** The Bi_3_TiNbO_9_ (BTNO) photocatalyst was prepared using a conventional solvothermal method. In a typical procedure, NaOH was dissolved in deionized water to prepare a 4.0 mol/L NaOH solution. Then, 3.0 mmol of Bi(NO_3_)_3_·5H_2_O was added to the solution under continuous stirring for 30 min. Subsequently, 0.5 mmol of Nb_2_O_5_ was thoroughly ground and introduced into the mixture, followed by another 30 min of stirring. After complete mixing, 1.0 mmol of titanium isopropoxide (C_16_H_36_O_4_Ti) was added dropwise under vigorous stirring to ensure homogeneity. The resulting suspension was transferred into a 100 mL Teflon-lined autoclave and heated at a ramp rate of 2 °C/min to 220 °C, which was maintained for 24 h. After the reaction, the system was allowed to cool naturally to room temperature. The product was collected and washed several times with deionized water and ethanol, followed by centrifugation and drying at 60 °C for 12 h to obtain the desired product, designated BTNO.

**Synthesis of Bi_2_MoO_6_:** Following a similar procedure, Bi_2_MoO_6_ (denoted as BMO) was also prepared via a solvothermal method. In brief, 2.0 mmol of Bi(NO_3_)_3_·5H_2_O and 1.0 mmol of Na_2_MoO_4_·2H_2_O were separately dissolved in 5.0 mL of ethylene glycol under ultrasonication for 30 min. The two solutions were then combined and stirred for 15 min. Subsequently, 20 mL of ethanol was added, and stirring continued for another 30 min. The homogeneous mixture was transferred into a 100 mL Teflon-lined autoclave and heated at 2 °C/min to 160 °C, holding for 12 h. After cooling to room temperature, the product was washed three times with deionized water and anhydrous ethanol, and dried in a vacuum oven at 60 °C for 12 h to obtain BMO.

**Figure 1:**
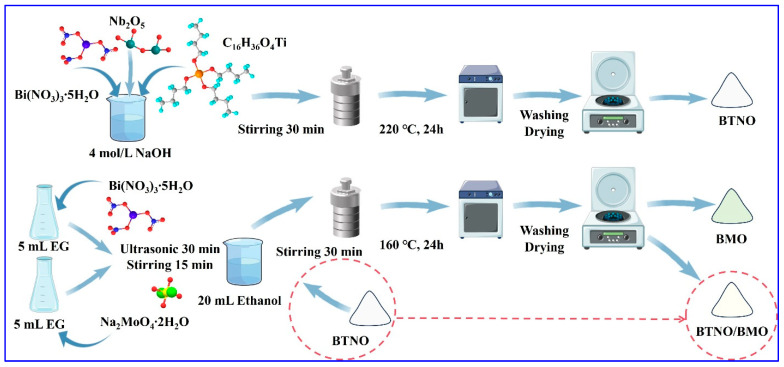
Schematic diagram of one-step, green synthesis for BTNO, BMO, and BTNO/BMO photocatalysts.

**Synthesis of BTNO/BMO:** The heterojunction was prepared based on the theoretical mass of BMO being 10% of the BTNO mass. To integrate the desired BTNO/BMO photocatalyst, 0.0485 g of Bi(NO_3_)_3_·5H_2_O and 0.0120 g of Na_2_MoO_4_·2H_2_O were separately dissolved in 5 mL of ethylene glycol under ultrasonication for 30 min. The two solutions were mixed and stirred for 15 min to yield Solution A. Meanwhile, 304.5 mg of as-synthesized BTNO was uniformly dispersed in 20 mL of ethanol via ultrasonication for 30 min, forming Solution B. Solution B was then poured into Solution A under stirring for 30 min. The mixture was transferred into a 100 mL Teflon-lined autoclave and reacted at 160 °C for 12 h. The final product was collected, washed three times with deionized water and ethanol, and dried under vacuum at 60 °C for 12 h. The obtained sample was labeled as BTNO/BMO. A schematic diagram of the synthesis process is illustrated in Figure 1.

### 2.2. Characterization

The morphological features of the as-prepared photocatalysts were examined by scanning electron microscopy (SEM, Hitachi S-3500 N, Hiatchi, Tokyo, Japan). Crystal structures were determined using X-ray diffraction (XRD) on a Bruker AXS D8 Advance X-ray diffractometer (Bruker, Billerica, MA, USA) with Cu-K*α* radiation, operating at a scanning rate of 5° min^−1^ over a 2θ range of 5–90°. X-ray photoelectron spectroscopy (XPS) measurements were conducted on a Thermo Scientific ESCALab250-Xi spectrometer (Thermo Fisher Scientific, Waltham, MA, USA) with monochromatic Al K*α* radiation (200 W) to analyze the surface chemical composition, electronic states, and valence band (VB) positions. All binding energies referred to the C 1s peak of adventitious carbon at 284.8 eV. Ultraviolet–visible diffuse reflectance spectra (UV-vis DRS) were recorded on a Shimadzu UV-2450 spectrophotometer equipped (Shimdazu, Kyoto, Japan) with an integrating sphere, using BaSO_4_ as a reflectance standard. For each measurement, an equal amount (30.0 mg) of the powder sample was uniformly mixed with BaSO_4_ for dilution.

### 2.3. Photocatalytic Activity Evaluation

The photocatalytic activity and versatile application potential of the as-synthesized catalysts were systematically evaluated through a series of reactions, including the degradation of tetracycline (TC) in aqueous solution, gaseous nitric oxide (NO) removal, and photocatalytic CO_2_ reduction. A 300 W xenon lamp (light intensity 250 mW/cm^2^, PLS-SXE300+, Perfect Light Source Co., Beijing, China) was employed as the light source for all tests, with specific configurations detailed for each reaction system.

The performance for degrading pollutants was first assessed using the typical antibiotic TC under visible-light irradiation. The Xe lamp was equipped with a 420 nm cutoff filter providing a light intensity of 320 mW·cm^−2^. In a typical procedure, a designated mass of the photocatalyst was added to 100.0 mL of an aqueous TC solution at a specific initial concentration. The suspension was first stirred in the dark for 30 min to establish adsorption–desorption equilibrium. Under continuous stirring, the mixture was then exposed to light. At given time intervals, about 5.0 mL of the suspension was sampled and centrifuged at 7500 rpm for 5 min to remove catalyst particles. The TC concentration in the clear supernatant was monitored by measuring its characteristic absorption peak at 356 nm using a UV-1800PC spectrophotometer (Shanghai Meixi Instrument Co., Ltd., Shanghai, China). The degradation efficiency (η) was calculated as η = (C_0_ − C)/C_0_ × 100% [31], where C_0_ and C are the initial and residual concentrations of TC, respectively. Recyclability tests were conducted to assess the stability of the optimal BTNO/BMO catalyst. After each photocatalytic degradation cycle, the catalyst was recovered by centrifugation, washed thoroughly with deionized water and ethanol, dried at 60 °C, and then reused under identical conditions for the next cycle. This process was repeated for three consecutive cycles.

The visible-light photocatalytic degradation activity was further evaluated by removing gaseous NO at ppb levels. Experiments were conducted in a continuous-flow setup featuring a custom quartz reactor and an online chemiluminescence NOx analyzer (Thermo Scientific, model 42i-DNMSDAA, range 0.0–2.0 ppm) with 40.0 mg of photocatalyst. During the tests, NO_2_ concentration changes were determined from the difference between NO and NOx. The NO removal efficiency (%) and NOx conversion rate (%) were calculated as follows [32]:

NO conversion (*φ*_NO_,%) = (1 − {[NO]_in_/[NO]_out_}) × 100%

NO_*x*_ conversion (*η*_NO*x*_,%) = ([NO*x*]_in_ − [NO*x*]_out_)/[NO_*x*_]_in_ × 100%

where [NO]_in_ and [NO]_out_ denote the inlet and outlet concentrations of nitrogen monoxide (in ppb), respectively, while [NOx]_in_, [NOx]_out_, and [NO_2_]_out_ denote the initial and final concentrations of nitrogen oxides (in ppb).

Photocatalytic CO_2_ reduction tests were performed in a Perfect Light Labsolar-6A reaction system, with the lamp positioned 4.0 cm from the reactor and circulating water for temperature control. Typically, 20.0 mg of photocatalyst was used. The reaction products were analyzed by an online GC7890A gas chromatograph (Agilent, Santa Clara, CA, USA) equipped with a thermal conductivity detector (TCD).

## 3. Results and Discussion

**Figure 2:**
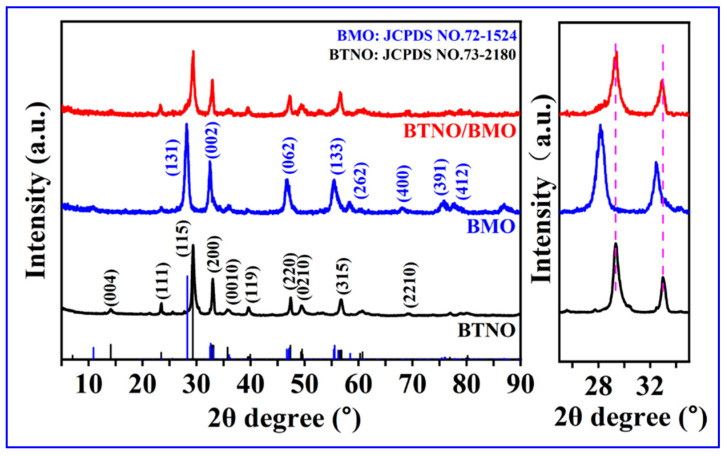
XRD patterns of as-obtained BTNO, BMO, and BTNO/BMO photocatalysts.

The crystal structures of the as-synthesized BTNO, BMO, and BTNO/BMO composite were first investigated by X-ray diffraction (XRD, Figure 2, left). The diffraction pattern of the pristine BTNO sample aligns well with the orthorhombic phase of BTNO (JCPDS No. 73-2180), exhibiting characteristic peaks corresponding to the (111), (115), (200), (0010), (220) and (315) planes, with no detectable impurity phases. Similarly, the pattern for pure BMO matches well with the standard card (JCPDS No. 72-1524), showing distinct peaks such as (131), (002), (062), and (133), which confirms its high purity. In the composite sample, where BTNO is the major component, the XRD pattern is dominated by the diffraction peaks of BTNO. However, a closer examination of the magnified region between 25° and 35° reveals slight but discernible shifts in the peak positions compared to pure BTNO (Figure 2, right). These shifts confirm the successful integration of BMO and the formation of the BTNO/BMO heterojunction, which likely induces lattice strain at the interface as further evidenced by calculated lattice strains in Table 1.

**Figure 3:**
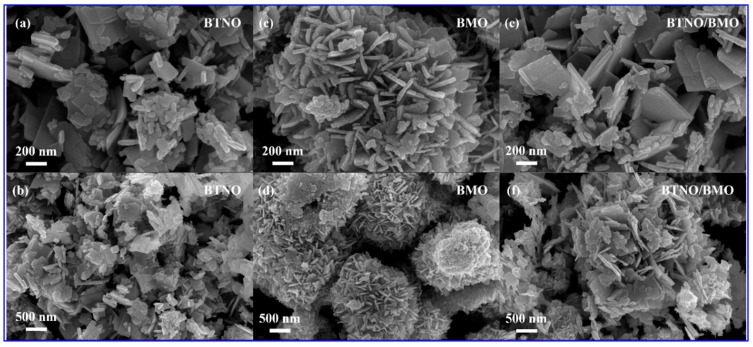
Representative SEM profiles showing the distinct morphologies of the as-obtained samples: (**a**,**b**) BTNO nanosheets; (**c**,**d**) BMO microspheres assembled from nanosheets; and (**e**,**f**) the closely integrated BTNO/BMO heterojunction.

The morphology/microstructure of the pristine BTNO, BMO, and as-integrated BTNO/BMO heterojunction were examined by scanning electron microscopy (SEM), as presented in Figure 3. The pure BTNO sample exhibits an architecture of irregularly stacked nanosheets with non-uniform sizes (Figure 3a,b). In contrast, pure BMO self-assembles into hierarchical microspheres composed of interconnected nanosheets (Figure 3c,d). As exhibited in Figure 3e,f, the as-integrated BTNO/BMO heterojunction reveals a closely integrated structure where the two components form a compact assembly of nanosheets. Notably, the nanosheets in the composite appear larger than those in pristine BTNO, and the overall packing is denser. This unique hierarchical architecture is advantageous for photocatalysis for the following reasons: (i) the nanosheet building blocks may provide a high specific surface area, facilitate reactant adsorption, and offer abundant active sites; (ii) the two-dimensional nature of nanosheets shortens the migration path for photogenerated charge carriers to the surface, thereby reducing the likelihood of bulk recombination. While the spherical superstructures of the BMO photocatalyst retain a high surface area, they also improve light-harvesting efficiency through enhanced light scattering and provide porous channels for mass transport [33]. However, it should be noted that excessive agglomeration could increase charge recombination and compromise photocatalytic performance. Hence, achieving optimal structural compactness is crucial.

**Figure 4:**
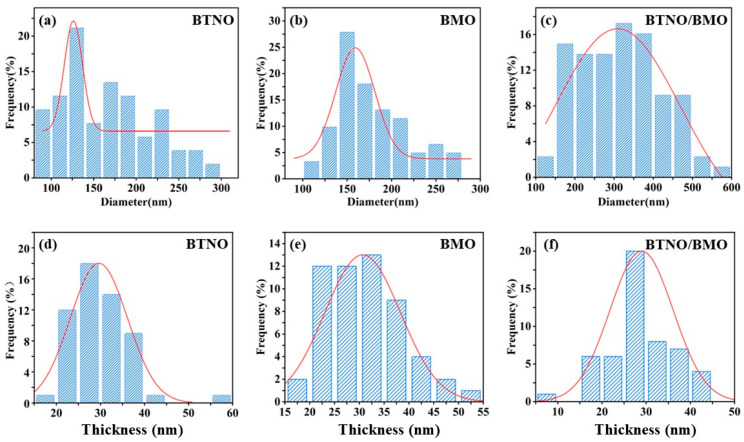
Statistical analysis of lateral size and thickness distributions for BTNO, BMO, and BTNO/BMO, revealing the formation of a heterojunction with a high aspect ratio. (**a**–**c**) Lateral size distributions of BTNO, BMO and BTNO/BMO, respectively; (**d**–**f**) Thickness distributions of BTNO, BMO and BTNO/BMO, respectively.

**Figure 5:**
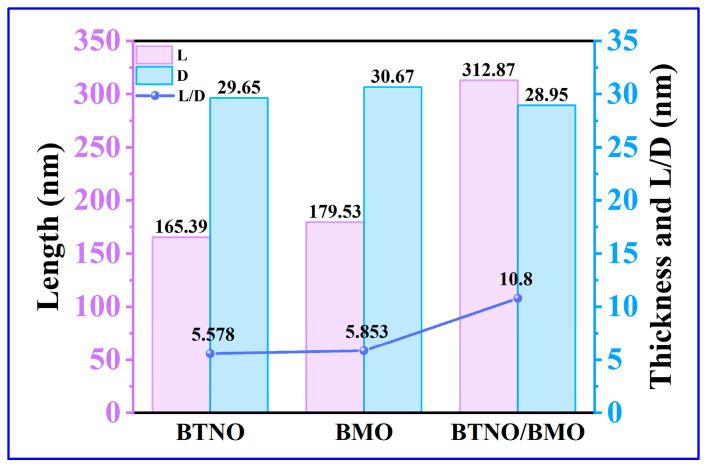
Changes in the length, thickness, and ratio of as-obtained BTNO, BMO and BTNO/BMO photocatalysts, respectively.

Furthermore, statistical analysis of the dimensions and thicknesses of the three materials was performed based on the SEM profiles. As illustrated in Figure 4, the average lateral size of the BTNO/BMO heterojunction increased to 312.87 nm, compared to 165.39 nm for BTNO and 179.53 nm for BMO. In contrast, the three photocatalysts exhibited similar thicknesses: BTNO, BMO and BTNO/BMO measured 29.65 nm, 30.67 nm and 28.95 nm, respectively. Consequently, the aspect ratio (the ratio of lateral size to thickness) of the composite increased significantly, from 5.58 for BTNO and 5.85 for BMO to 10.80 for the heterojunction (Figure 5). This elevated aspect ratio is expected to enhance photon capture, establish more efficient charge transport pathways, and suppress the electron–hole recombination, thereby supplying more energy for photocatalytic reactions.

**Figure 6:**
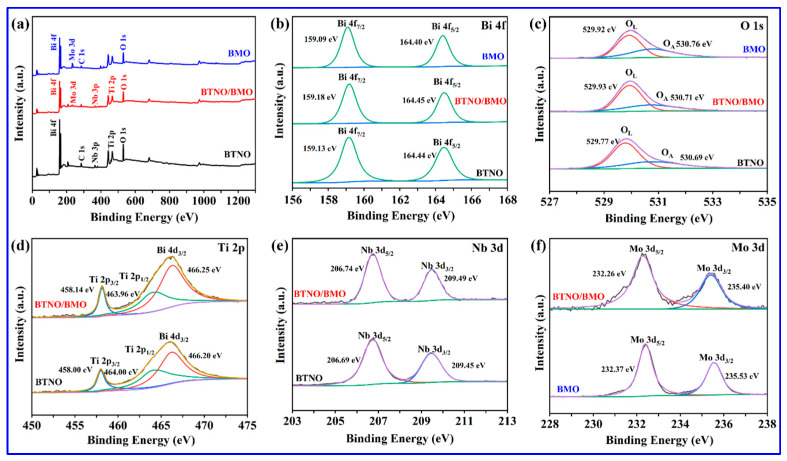
XPS characterization confirming interfacial charge transfer: (**a**) the survey spectra and (**b**–**f**) high-resolution spectra of key elements Bi 4f, O 1s, Ti 2p, Nb 3d, Mo 3d for BTNO, BMO, and as-integrated BTNO/BMO heterojunction.

To elucidate the elemental composition, chemical states, and interfacial interactions in the pure-phase and as-integrated heterojunction, X-ray photoelectron spectroscopy (XPS) characterization was conducted. Three representative survey spectra in Figure 6a confirm the presence of all expected elements, e.g., Bi, O, Ti, and Nb for BTNO, and Bi, O, and Mo for BMO in their respective samples, with no detectable impurities aside from the adventitious C 1s used for calibration. Moreover, the coexistence of characteristic signals from both BTNO and BMO in the composite’s survey spectrum verifies the successful preparation of the BTNO/BMO heterojunction. High-resolution spectra provide further insight into the chemical environment. As shown in Figure 6b, the Bi 4f spectra of pristine BTNO exhibit doublet peaks located at 159.13 eV and 164.44 eV corresponding to Bi 4f_7/2_ and Bi 4f_5/2_ of Bi^3+^, respectively. In comparison, in the Bi 4f spectrum of BMO, two observed peaks at 159.09 eV and 164.40 eV correspond to the Bi 4f_7/2_ and Bi 4f_5/2_ states of Bi^3+^, respectively. Interestingly, the Bi 4f peaks shift to higher binding energies upon heterojunction formation. In the O 1s region shown in Figure 6c, the spectrum of BTNO was deconvoluted into lattice oxygen (O_L_) at 529.77 eV and adsorbed oxygen species (O_A_) at 530.69 eV. For BMO, the O 1s peaks at 529.92 eV and 530.76 eV are assigned to lattice oxygen and surface hydroxyl groups, respectively. After the formation of a heterojunction, the O 1s peaks shift to lower binding energies compared to the two monomers.

The shifts in core-level binding energies are more pronounced in the cationic species (Figure 6d,e). In the as-integrated BTNO/BMO heterojunction, the Ti 2p spectrum with dominant peaks at 458.00 eV and 464.00 eV (corresponding to the Ti 2p_3/2_ and Ti 2p_1/2_ states) and the Nb 3d spectrum with observed peaks at 206.69 eV and 209.45 eV (attributed to the Nb 3d_5/2_ and Nb 3d_3/2_ states) shift positively compared to their positions in pristine BTNO [34,35]. Conversely, in the Mo 3d spectrum in Figure 6f, the Mo 3d_5/2_ at 232.37 eV and Mo 3d_3/2_ at 235.53 eV, characteristic of Mo^6+^, shift negatively in the composite [36,37]. This systematic and opposite shifting trend, where the binding energies of BTNO constituents (Bi, Ti, and Nb) increase while those of BMO composites (Bi and Mo) decrease, provides strong evidence for interfacial electron transfer. The increased binding energy suggests a decreased electron density around the atoms in BTNO, whereas the decreased binding energy indicates an increased electron density in BMO. This observation confirms that electrons migrate from the BTNO to BMO across the heterojunction interface. Such charge redistribution facilitates the more efficient separation of photogenerated electron–hole pairs and enhances charge carrier transport, which is crucial for boosting photocatalytic performance.

**Figure 7:**
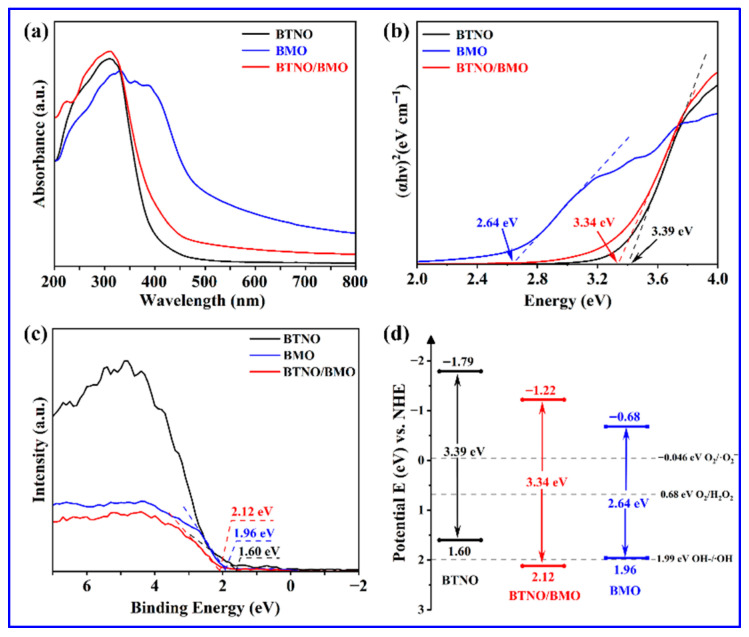
Optical properties and electronic band structures analysis: (**a**) UV-Vis DRS; (**b**) corresponding Tauc plots; (**c**) XPS valence band measurements; and (**d**) schematic illustration of the energy band alignment for BTNO, BMO, and BTNO/BMO photocatalysts.

Figure 7 presents the UV-vis diffuse reflectance spectra (DRS), corresponding Tauc plots for bandgap estimation, and the derived energy band structure diagrams for the pristine BTNO, BMO, and BTNO/BMO heterojunction. As shown in Figure 7a, the absorption edge of BTNO is located around 400 nm, with its absorption predominantly in the ultraviolet region, characteristic of a wide-bandgap semiconductor. In contrast, pure BMO exhibits a significantly red-shifted absorption edge near 530 nm, affirming its superior visible-light harvesting capability attributable to a narrower bandgap. The absorption profile of the BTNO/BMO heterojunction demonstrates a composite characteristic of its constituent phases. Its absorption intensity in the 200–400 nm range is enhanced compared to pristine BTNO, and its absorption edge resides between those of BTNO and BMO [38]. The optical bandgap energies (E_g_) were determined from the empirical formula *α*h*ν* = A(h*ν* − E_g_)^n^, where *α*, h, *ν*, A, and E_g_ represent the absorption coefficient, Planck’s constant, optical frequency, proportionality constant, and bandgap energy, respectively. The exponent n depends on the nature of the optical transition, with *n* = 1/2 for direct and *n* = 2 for indirect bandgap semiconductors [19]. As shown in Figure 7b, the calculated bandgaps for BTNO, BMO, and the heterojunction composite BTNO/BMO are 3.39 eV, 2.64 eV, and 3.34 eV, respectively. The intermediate bandgap of the heterojunction indicates effective band structure modulation, likely induced by interfacial charge transfer and electronic coupling between the two components.

Combining the bandgap values with the valence band (VB) positions obtained from XPS analysis (Figure 7c), the conduction band (CB) potentials were deduced, thereby constructing a schematic energy band diagram (Figure 7d). The respective CB and VB positions are estimated as −1.79 eV and 1.60 eV for the BTNO sample, −0.68 eV and 1.96 eV for the BMO photocatalyst, and −1.22 eV and 2.12 eV for the BTNO/BMO heterojunction. This band alignment not only facilitates the directed migration of photogenerated charge carriers but also allows for a preliminary assessment of the types of reactive oxygen species that can be generated based on their standard redox potential.

**Figure 8:**
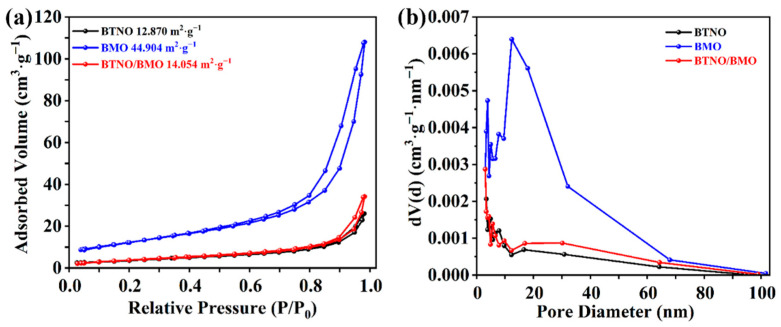
Comparative analysis of the porous texture: (**a**) N_2_ adsorption–desorption isotherms and (**b**) pore size distribution for pristine BTNO, BMO, and as-integrated BTNO/BMO heterojunction.

The specific surface areas and porosity of the pristine samples and as-integrated BTNO/BMO heterojunction were investigated by N_2_ adsorption–desorption measurements (Figure 8). All the samples exhibit Type-IV isotherms with distinct H3-type hysteresis loops according to the IUPAC classification [39], indicating the presence of mesoporous structures. The measured specific surface areas for the BTNO, BMO, and as-integrated BTNO/BMO heterojunction are 12.870 m^2^·g^−1^, 44.904 m^2^·g^−1^, and 14.054 m^2^·g^−1^, respectively. The significantly larger surface area of pristine BMO is consistent with its three-dimensional hierarchical architecture assembled from nanosheets. Notably, the surface area of the heterojunction composite is higher than that of pristine BTNO. The remarkable increase in the surface area can be attributed to the introduction of BMO and the potential formation of a more open, interconnected porous network during the heterostructure assembly process. A larger specific surface area generally provides more active sites for the adsorption of reactant molecules and surface reactions, which is a beneficial factor for enhancing photocatalytic efficiency [40].

The photocatalytic performances of as-synthesized photocatalysts were systematically evaluated via oxidative degradation of organic/gaseous pollutants and reductive energy conversion, to reveal wide-ranging utility of the as-integrated BTNO/BMO heterojunction.

**Figure 9:**
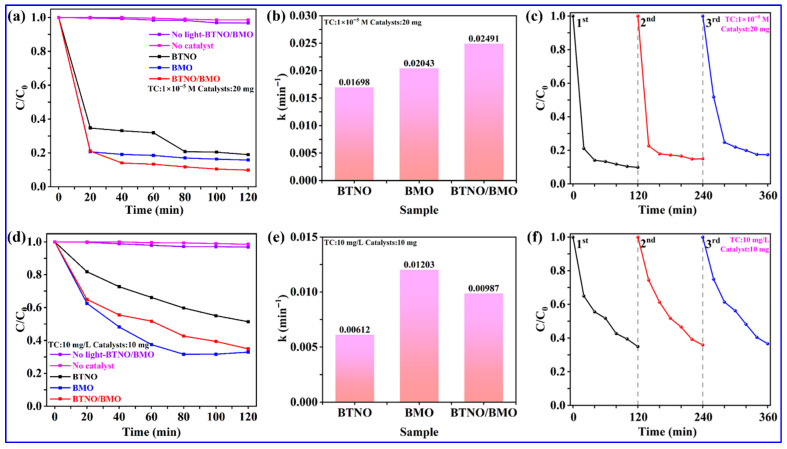
Photocatalytic degradation of TC under different conditions: (**a**,**b**) degradation profiles and kinetic fitting curves for a 1 × 10^−5^ M TC solution; (**d**,**e**) degradation profiles and kinetic fitting curves for a 10.0 mg/L TC solution. Recyclability of the BTNO/BMO heterojunction for degradation of (**c**) 1 × 10^−5^ M and (**f**) 10.0 mg/L of TC under visible-light irradiation over three consecutive cycles, respectively.

The photocatalytic activities of the pristine BTNO, BMO, and BTNO/BMO heterojunction were first evaluated by monitoring the degradation of a typical antibiotic, tetracycline (TC), under visible-light irradiation. The TC decomposition process was monitored by measuring the characteristic absorption of TC at its maximum wavelength of 356 nm using UV-Vis spectroscopy (Figure 9). Based on the results of the blank control group experiment, degradation under conditions without a catalyst or without light exposure is negligible. Figure 9a displays the photocatalytic degradation of a 1 × 10^−5^ M TC solution using 20.0 mg of each photocatalyst. All samples exhibited significant visible-light performances, with rapid TC degradation occurring within the initial 20 min. After 120 min of visible-light illumination, the degradation efficiencies for BTNO, BMO, and BTNO/BMO reached 81.1%, 84.3%, and 90.2%, respectively. The superior performance of the as-integrated heterojunction might be ascribed to the effective interfacial coupling with unique BMO, which extends light absorption and, more critically, facilitates the spatial separation and migration of photogenerated charge carriers.

The reaction kinetic constant *k* was further analyzed by fitting the data to a pseudo-first-order model: ln(C_0_/C) = *k*t. As shown in the corresponding plot, the BTNO/BMO composite exhibited the steepest slope, corresponding to the highest apparent rate constant *k* for visible-light TC decomposition. The accurate *k* values were calculated to be 0.01698 min^−1^ for BTNO, 0.02043 min^−1^ for BMO, and 0.02491 min^−1^ for BTNO/BMO, respectively (Figure 9b). To further probe the structure–activity relationship, visible-light TC degradation was also examined by dispersing 10.0 mg of catalyst powder in a 10 mg/L TC aqueous solution (Figure 9d). After 120 min of visible-light illumination, the TC degradation efficiencies were 48.7% for BTNO, 67.1% for BMO, and 65.1% for the BTNO/BMO heterojunction, respectively. Correspondingly, the kinetic analysis revealed that pristine BMO (*k* = 0.01203 min^−1^) slightly outperformed the heterojunction with a *k* value of 0.00987 min^−1^, while BTNO remained the least active with a *k* of 0.00162 min^−1^ (Figure 9e). This shift in visible-light TC degradation performance highlights the influence of reaction conditions on the dominant factors governing photocatalytic efficiency. At a high catalyst-to-pollutant ratio (20.0 mg catalyst, 1 × 10^−5^ M TC), active sites are abundant. In this system, the enhanced intrinsic activity of each site in the heterojunction, due to improved charge separation, dictates the overall superior performance [41]. However, under a low catalyst-to-pollutant ratio with 10.0 mg of catalyst in 10 mg/L of TC, mass transfer efficiency may become the limiting factor. The potentially more complex pore structure and diffusion pathways within the heterojunction composite could hinder the access of abundant TC molecules to all active sites compared to the simpler BMO monomer, thereby explaining the marginally lower activity of the composite in this specific scenario [42].

To evaluate the stability of the synthesized BTNO/BMO heterojunction, we performed three consecutive photocatalytic degradation cycles under visible-light irradiation, using TC as the model pollutant at different concentrations of 1 × 10^−5^ M and 10 mg/L. After each cycle, the catalyst was recovered by centrifugation, washed with deionized water and ethanol, and dried before reuse under identical reaction conditions. As shown in Figure 9c,f, the heterojunction exhibited excellent recyclability. After three cycles, the TC degradation efficiency for the 1 × 10^−5^ M solution decreased only from 90.2% to 82.7% (a loss of 7.5%), while for the 10 mg/L solution, it decreased marginally from 65.1% to 63.4% (a loss of merely 1.7%). This minimal activity loss demonstrates the high structural and chemical stability of the BTNO/BMO heterojunction under repeated photocatalytic operation.

Furthermore, porosity plays a decisive role under different catalyst-to-pollutant ratios. Under high catalyst loading, e.g., 20.0 mg of catalyst and low pollutant concentration, active sites are abundant and mass transfer is not limited. In this regime, enhanced charge separation in the heterojunction dominates the photocatalytic activity. Conversely, under low catalyst loading, e.g., 10.0 mg of catalyst and high pollutant concentration, reactant accessibility becomes critical. Although the heterojunction possesses a moderate surface area, its pore architecture may impose diffusion limitations compared to the highly porous BMO microspheres. This explains the observed performance shift in tetracycline degradation (Figure 9), where BMO slightly outperformed the heterojunction under low-catalyst conditions, whereas the heterojunction exhibited superior activity under high-catalyst conditions. Therefore, optimizing both porosity for mass transfer and interfacial charge separation for carrier utilization is essential for designing versatile photocatalysts adaptable to varying pollutant loads.

Additionally, as shown in Table 2 below, the TC degradation performance of the synthesized BTNO/BMO heterojunction material was compared with several other reported heterojunction materials [4,43,44,45,46,47,48,49,50]. It can be observed that this BTNO/BMO composite not only demonstrates competitive degradation efficiency but also exhibits comparable or superior cycling stability, highlighting its potential as a robust photocatalyst for environmental remediation.

**Figure 10:**
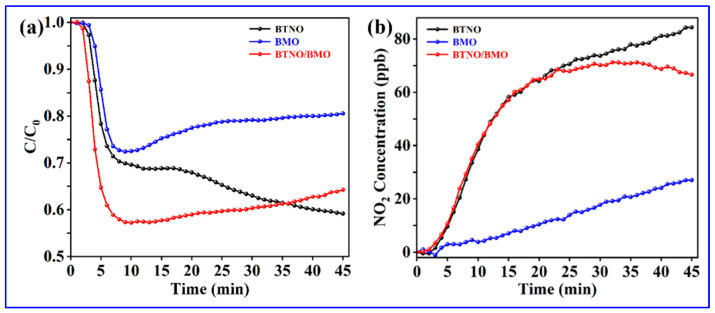
(**a**) Photocatalytic NO removal efficiency and (**b**) toxic product NO_2_ yields in the presence of as-obtained BTNO, BMO, and BTNO/BMO heterojunction photocatalysts.

To validate the wide range implementations of as-synthesized photocatalysts, the visible-light photocatalytic oxidation performance was assessed by removing gaseous nitric oxide (NO) at parts-per-billion (ppb) levels. As shown in Figure 10, both BTNO and the as-integrated BTNO/BMO heterojunction exhibited superior NO conversion efficiency compared to pristine BMO. Initially, the BTNO/BMO heterojunction demonstrated the best NO removal performance. However, the removal rate began to decline after 15 min and eventually decreased below that of pristine BTNO by the end of the test period. We assumed that the accumulation of NO upon prolonged illumination appeared to induce catalyst deactivation resulting in decreased NO removal. Regarding byproduct generation, NO_2_ was maintained below 90 ppb for BTNO, 30 ppb for BMO, and 70 ppb for the BTNO/BMO heterojunction, confirming the low production of toxic byproducts across all samples [51]. The construction of the heterojunction by growing BMO on BTNO nanosheets presents significant potential for environmental remediation. Future work will be focused on elucidating the degradation pathways and further improving the catalyst’s stability under long-term operation.

**Figure 11:**
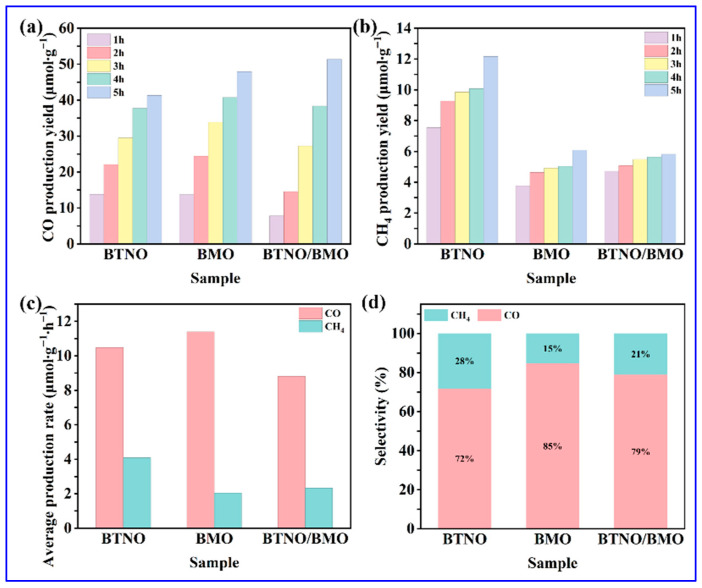
Visible light Driven photocatalytic CO_2_ reduction performance: (**a**,**b**) Time-dependent CO and CH_4_ evolution, (**c**) average production rates over 5 h visible-light illumination, and (**d**) CO_2_ reduction product selectivity for BTNO, BMO, and BTNO/BMO photocatalyst, respectively.

Finally, the visible-light photocatalytic capability of as-obtained samples was evaluated via CO_2_ reduction. As shown in Figure 11a,b, the yields of CO and Methane (CH_4_) for all photocatalysts increased gradually over the 5 h visible-light irradiation period. It can be noticed that the CO yield showed a particularly pronounced upward trend. After 5 h, the total CO yields for BTNO, BMO, and BTNO/BMO reached 41.3 μmol·g^−1^, 47.9 μmol·g^−1^, and 51.3 μmol·g^−1^, respectively. In contrast, the increase in CH_4_ yield was more modest, with final amounts of 12.1 μmol·g^−1^ for BTNO, 6.1 μmol·g^−1^ for BMO, and 5.8 μmol·g^−1^ for BTNO/BMO, respectively. The average production rates, calculated over the 5 h period, provide further insight into catalytic behavior (Figure 11c). For CO production, pristine BMO exhibited the highest average rate of 11.39 μmol·g^−1^·h^−1^, followed by BTNO with 10.47 μmol·g^−1^·h^−1^, with the as-integrated BTNO/BMO heterojunction showing a slightly lower rate of 8.80 μmol·g^−1^·h^−1^, respectively. Conversely, for CH_4_ production, BTNO demonstrated the highest average rate with 4.08 μmol·g^−1^·h^−1^, while the heterojunction displayed 2.33 μmol·g^−1^·h^−1^ yield, slightly outperforming pristine BMO, which exhibited 2.04 μmol·g^−1^·h^−1^ CH_4_ production. As summarized in Figure 11d, all catalysts exhibited high selectivity towards CO, with the heterojunction’s selectivity intermediate between the two pristine materials, consistent with its composite nature [52]. The distinct product distribution suggests that the interfacial engineering in BTNO/BMO modulates the reaction pathway and energy barriers for different reduction products.

**Figure 12:**
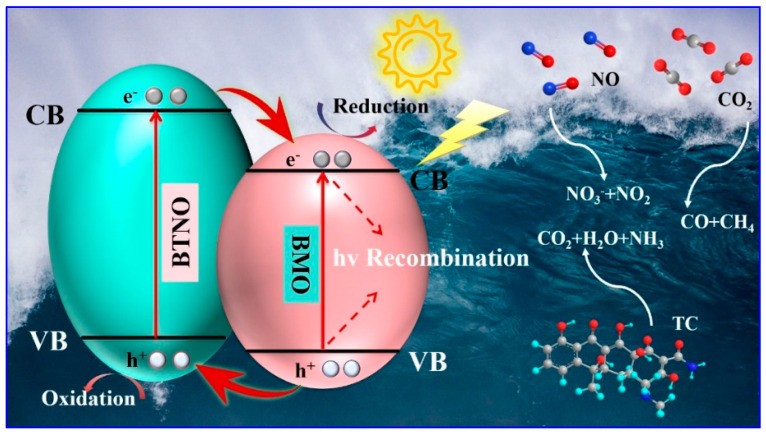
Schematic diagram of the visible-light photocatalytic mechanism of BTNO/BMO heterojunction composites in tetracycline degradation, nitric oxide removal, and carbon dioxide reduction.

Based on the foregoing analysis, a reaction mechanism for the enhanced photocatalytic performance of the as-integrated BTNO/BMO heterojunction is proposed (Figure 12). This mechanism accounts for the efficacy of photocatalysts in diverse applications including aquatic pollutant degradation and atmospheric hazardous remediation. The coupling of energy bands at the heterojunction interface modifies the original band alignments, effectively widening the composite’s responsive energy landscape. Under visible-light irradiation, electrons in BTNO are excited from the valence band to the conduction band. The intimate contact between BTNO and BMO facilitates the formation of a Type-II heterostructure. This configuration drives the transfer of photoinduced electrons from the conduction band of BTNO to that of BMO, while simultaneously directing holes from the valence band of BMO to that of BTNO. This spatial charge separation effectively suppresses carrier recombination, thereby enhancing the utilization efficiency of photogenerated charge carriers. The separated charges subsequently initiate surface redox reactions. The accumulated electrons and holes promote the generation of photocatalytically active oxygen species, which through synergistic action, progressively decompose complex antibiotic molecules into simpler organic intermediates, ultimately achieving complete mineralization into CO_2_, H_2_O, and inorganic ions. Furthermore, the electrons facilitate the reduction of stable CO_2_ molecules to CO and CH_4_, while the holes contribute to the oxidation of NO, primarily to nitrate (NO_3_^−^) with trace amounts of NO_2_.

## 4. Conclusions

In summary, a novel BTNO/BMO heterojunction composite was successfully constructed via a solvothermal method and demonstrated high-performance photocatalytic activity for environmental purification. The composite effectively integrates the structural and optical advantages of its components, exhibiting a widened light absorption range and enhanced charge separation efficiency compared to the pristine materials. The photocatalytic performance was systematically evaluated across both oxidation and reduction processes. For oxidation reactions, the heterojunction showed superior activity in the degradation of aqueous tetracycline and the removal of gaseous NO. For reduction reactions, it facilitated the conversion of CO_2_ into solar fuels, primarily CO. The significantly enhanced activity across these diverse reactions is primarily attributed to the formation of a Type-II heterostructure, which drives the spatial separation of photogenerated electron–hole pairs, thereby increasing the availability of charge carriers for surface redox reactions. This work provides valuable insights into the design of efficient, dual-functional photocatalysts capable of managing both oxidative pollutant degradation and reductive energy conversion, offering a promising strategy for addressing intertwined environmental and energy challenges.

## Figures and Tables

**Table 1:** Crystalline cell parameters corresponding to XRD of the samples.

Sample	*a*	*b*	*c*
BTNO	5.41628 Å	5.42794 Å	25.16998 Å
BMO	5.44178 Å	5.43465Å	16.51194 Å
BTNO/BMO	5.40203 Å	5.43261 Å	25.28395 Å

**Table 2:** The state-of-the-art of heterojunction photocatalysts and their implementation for photocatalytic pollutant degradations (TC: tetracycline, CIP: ciprofloxacin, RhB: Rhodamine B).

Photocatalyst	Light Source	Catalyst Dosage	Pollutants (Concentration)	Degradation (%)	Ref.
BiOIO_3_/BiOBr	12 W LED lamp	80 mg	TC (20 mg/L)	74.91% in 80 min	[43]
CuFe_2_O_4_/Bi_2_MoO_6_	300 W Xe lamp	30 mg	TC (50 mg/L)	94.02% in 30 min	[4]
Bi_2_S_3_/BiFeO_3_	300 W Xe lamp, λ > 420 nm	20 mg	TC (20 mg/L)	74.00% in 120 min	[44]
FeIn_2_S_4_/BiOBr	500 W Xe lamp, λ > 400 nm	30 mg	CIP (5 mg/L)	94.10% in 120 min	[45]
			TC (15 mg/L)	89.20% in 100 min	
BiOI/BiVO_4_	500 W Xe lamp, λ > 420 nm	50 mg	RhB (10 mg/L)	~100.00% in 4 h	[46]
			TC (20 mg/L)	~65.00% in 20 min	
Ag_2_S/TiO_2_	100 W Xe lamp	200 mg	TC (10 mg/L)	72.3% in 80 min	[47]
In_2_O_3_/Bi_2_WO_6_	300 W Xe lamp, λ > 400 nm	20 mg	TC (20 mg/L)	86.00% in 70 min	[48]
MoS_2_/BiVO_4_	300 W Xe lamp, λ > 420 nm	50 mg	TC (5 mg/L)	93.70% in 90 min	[49]
g-C_3_N_4_/TiO_2_	Xe arc lamp, λ > 420 nm	20 mg	TC (10 mg/L)	65.00% in 30 min	[50]
Bi_3_TiNbO_9_/Bi_2_MoO_6_	300 W Xe lamp, λ > 420 nm	10 mg	TC (10 mg/L)	65.10% in 120 min	This work
		20 mg	TC (10^−5^ mol/L)	90.20% in 120 min	

## Data Availability

The raw data supporting the conclusions of this article will be made available by the authors on request.
